# 1,8-Bis(benz­yloxy)-3,6-diiodo­naphthalene

**DOI:** 10.1107/S1600536810019355

**Published:** 2010-06-05

**Authors:** Ying Liu, Leyong Wang, Jingjing Wang, Li Liu, Mingyu Teng

**Affiliations:** a School of Chemistry and Chemical Engineering, Nanjing University, Hankou Road 22, Nanjing 210093, People’s Republic of China

## Abstract

In the crystal structure of the title compound, C_24_H_18_I_2_O_2_, one benzene ring is almost coplanar with the naphthyl system [dihedral angle = 6.6 (4)°], whereas the other is almost orthogonal [73.1 (2)°]. The crystal structure is consolidated by C—H⋯O and C—H⋯π inter­actions.

## Related literature

For biomarkers for the Melanin metabolic process, see: Minto & Townsend (1997 ▶); Thompson *et al.* (2000 ▶); Zhang *et al.* (2008 ▶). For the synthesis of the title compound, see: Paruch *et al.* (2000 ▶).
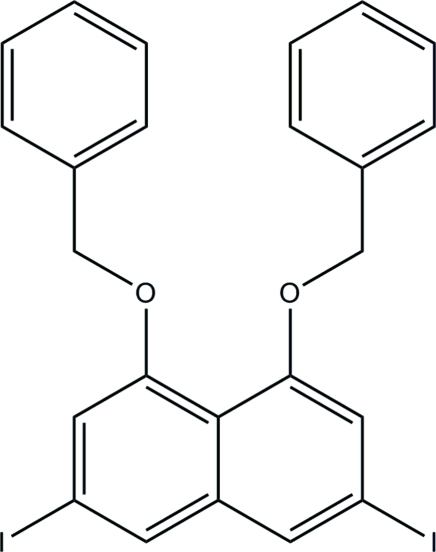


## Experimental

### 

#### Crystal data

C_24_H_18_I_2_O_2_
*M* *_r_* = 592.18
Monoclinic, 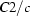

*a* = 31.222 (4) Å
*b* = 5.5684 (8) Å
*c* = 27.445 (4) Å
β = 118.680 (2)°
*V* = 4186.1 (10) Å^3^
*Z* = 8
Mo *K*α radiation
μ = 3.02 mm^−1^
*T* = 291 K
0.28 × 0.24 × 0.22 mm

#### Data collection

Bruker SMART APEX CCD diffractometer
Absorption correction: multi-scan (*SADABS*; Sheldrick, 2004 ▶) *T*_min_ = 0.485, *T*_max_ = 0.556
10700 measured reflections
4111 independent reflections
2679 reflections with *I* > 2σ(*I*)
*R*_int_ = 0.038

#### Refinement

*R*[*F* ^2^ > 2σ(*F* ^2^)] = 0.052
*wR*(*F* ^2^) = 0.101
*S* = 1.03
4111 reflections
253 parameters
H-atom parameters constrained
Δρ_max_ = 0.81 e Å^−3^
Δρ_min_ = −0.86 e Å^−3^

### 

Data collection: *SMART* (Bruker, 2001 ▶); cell refinement: *SAINT* (Bruker, 2001 ▶); data reduction: *SAINT*; program(s) used to solve structure: *SHELXTL* (Sheldrick, 2008 ▶); program(s) used to refine structure: *SHELXTL*; molecular graphics: *SHELXTL*; software used to prepare material for publication: *SHELXTL*.

## Supplementary Material

Crystal structure: contains datablocks I, global. DOI: 10.1107/S1600536810019355/tk2673sup1.cif

Structure factors: contains datablocks I. DOI: 10.1107/S1600536810019355/tk2673Isup2.hkl

Additional supplementary materials:  crystallographic information; 3D view; checkCIF report

## Figures and Tables

**Table 1:** Hydrogen-bond geometry (Å, °) *Cg* is the centroid of the C19*A*–C24*A* ring.

*D*—H⋯*A*	*D*—H	H⋯*A*	*D*⋯*A*	*D*—H⋯*A*
C24*A*—H24*A*⋯O1*BA*^i^	0.93	2.49	3.348 (8)	154
C18*A*—H18*A*⋯*Cg*^ii^	0.97	2.77	3.513 (5)	134

Symmetry codes: (i) 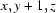; (ii) 
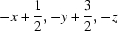
.

**Fig. 1.**
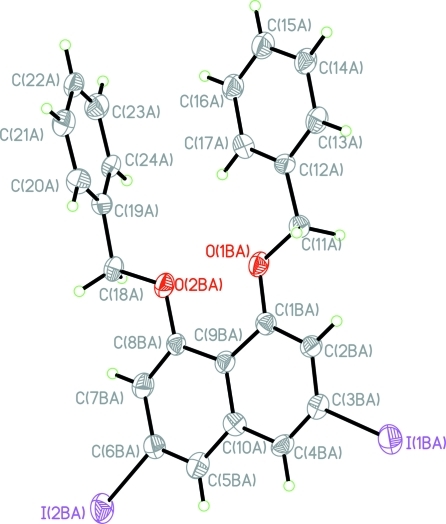
The molecular structure of (I), with atom labels and 40% probability displacement ellipsoids for non-H atoms.

## supplementary crystallographic information

### Comment

The title compound (I), a known compound (Paruch *et al.*, 2000), was obtained as an intermediate during deuterium substitution reactions for generating biomarkers for the melanin metabolic process (Minto & Townsend, 1997; Thompson *et al.*, 2000; Zhang *et al.*, 2008). In order to reduce steric congestion, the benzene rings have different orientations with respect to the central naphthyl ring. Thus, one benzene ring (C12A–C17A) is almost co-planar with the naphthyl ring [dihedral angle = 6.6 (4)°] whereas the other (C19A–C24A) is almost orthogonal [dihedral angle = 73.1 (2)°].

Molecules are linked via weak intermolecular C–H···O [C24A–H24A···O1BA^i^ = 2.49 Å, C24A···O1BA^i^ = 3.348 (8) Å with angle at H24A = 15° for i: x, 1+y, z] and C–H···π [C18A–H18A···Cg(C19A–C24A)^ii^ = 2.77 Å, C18A···Cg(C19A–C24A)^ii^ = 3.513 (5) Å with angle at H = 134° for ii: 1/2-x, 3/2-y, -z] interactions.

### Experimental

The precursor, 3,6-diiodonaphthalene-1,8-diol (0.4 g, 0.97 mmol), was added to a mixture of (bromomethyl)benzene (0.5 g, 2.92 mmol), potassium carbonate (0.53 g, 3.84 mmol), and acetone (40 mL) in a 50 mL flask. The mixture was heated to reflux for 4.5 hours and the solvent removed. The crude product was purified by column chromatography to give the pure title compound (I). The single crystals were obtained by slowly evaporating the solution of (I) from a petroleum and ethyl acetate mixture solvent.

### Refinement

All the H atoms were positioned geometrically and refined using a riding model with C—H = 0.93–0.97 Å, and with *U*_iso_(H) = 1.2*U*_eq_(C).

### Crystal data

**Table:**

C_24_H_18_I_2_O_2_	*F*(000) = 2272
*M**_r_* = 592.18	*D*_x_ = 1.879 Mg m^−^^3^
Monoclinic, *C*2/*c*	Mo *K*α radiation, λ = 0.71073 Å
Hall symbol: -C 2yc	Cell parameters from 3618 reflections
*a* = 31.222 (4) Å	θ = 2.6–27.6°
*b* = 5.5684 (8) Å	µ = 3.02 mm^−^^1^
*c* = 27.445 (4) Å	*T* = 291 K
β = 118.680 (2)°	Block, brown
*V* = 4186.1 (10) Å^3^	0.28 × 0.24 × 0.22 mm
*Z* = 8	

### Data collection

**Table:**

Bruker SMART APEX CCD diffractometer	4111 independent reflections
Radiation source: sealed tube	2679 reflections with *I* > 2σ(*I*)
graphite	*R*_int_ = 0.038
φ and ω scans	θ_max_ = 26.0°, θ_min_ = 1.7°
Absorption correction: multi-scan (*SADABS*; Sheldrick, 2004)	*h* = −38→36
*T*_min_ = 0.485, *T*_max_ = 0.556	*k* = −6→5
10700 measured reflections	*l* = −31→33

### Refinement

**Table:**

Refinement on *F*^2^	Primary atom site location: structure-invariant direct methods
Least-squares matrix: full	Secondary atom site location: difference Fourier map
*R*[*F*^2^ > 2σ(*F*^2^)] = 0.052	Hydrogen site location: inferred from neighbouring sites
*wR*(*F*^2^) = 0.101	H-atom parameters constrained
*S* = 1.03	*w* = 1/[σ^2^(*F*_o_^2^) + (0.04*P*)^2^ + 1.22*P*] where *P* = (*F*_o_^2^ + 2*F*_c_^2^)/3
4111 reflections	(Δ/σ)_max_ < 0.001
253 parameters	Δρ_max_ = 0.81 e Å^−^^3^
0 restraints	Δρ_min_ = −0.86 e Å^−^^3^

### Special details

**Table:**

Geometry. All esds (except the esd in the dihedral angle between two l.s. planes) are estimated using the full covariance matrix. The cell esds are taken into account individually in the estimation of esds in distances, angles and torsion angles; correlations between esds in cell parameters are only used when they are defined by crystal symmetry. An approximate (isotropic) treatment of cell esds is used for estimating esds involving l.s. planes.
Refinement. Refinement of *F*^2^ against ALL reflections. The weighted *R*-factor wR and goodness of fit *S* are based on *F*^2^, conventional *R*-factors *R* are based on *F*, with *F* set to zero for negative *F*^2^. The threshold expression of *F*^2^ > σ(*F*^2^) is used only for calculating *R*-factors(gt) etc. and is not relevant to the choice of reflections for refinement. *R*-factors based on *F*^2^ are statistically about twice as large as those based on *F*, and *R*- factors based on ALL data will be even larger.

### Fractional atomic coordinates and isotropic or equivalent isotropic displacement parameters (Å^2^)

**Table:**

	*x*	*y*	*z*	*U*_iso_*/*U*_eq_	
I1BA	0.532315 (16)	−0.52925 (9)	0.184014 (16)	0.05132 (16)	
I2BA	0.419035 (16)	0.51148 (9)	−0.067214 (17)	0.05151 (15)	
O2BA	0.35208 (14)	0.3984 (7)	0.08149 (16)	0.0368 (9)	
O1BA	0.39304 (14)	0.1087 (7)	0.16410 (16)	0.0363 (9)	
C7BA	0.3862 (2)	0.4364 (11)	0.0199 (2)	0.0382 (14)	
H7AA	0.3664	0.5679	0.0024	0.046*	
C1BA	0.42086 (19)	0.0189 (11)	0.1420 (2)	0.0324 (12)	
C18A	0.31949 (19)	0.5945 (10)	0.0502 (2)	0.0328 (12)	
H18A	0.3029	0.5558	0.0109	0.039*	
H18B	0.3382	0.7401	0.0554	0.039*	
C9BA	0.41640 (19)	0.1355 (11)	0.0930 (2)	0.0320 (12)	
C8BA	0.38423 (18)	0.3302 (10)	0.0639 (2)	0.0295 (12)	
C3BA	0.4817 (2)	−0.2471 (10)	0.1437 (2)	0.0344 (13)	
C12A	0.36459 (19)	0.1100 (11)	0.2302 (2)	0.0319 (12)	
C16A	0.3090 (2)	0.4135 (12)	0.2268 (2)	0.0433 (15)	
H16A	0.2918	0.5539	0.2108	0.052*	
C24A	0.28382 (19)	0.8405 (11)	0.0981 (2)	0.0327 (13)	
H24A	0.3086	0.9526	0.1070	0.039*	
C10A	0.4473 (2)	0.0466 (11)	0.0720 (2)	0.0387 (14)	
C20A	0.2453 (2)	0.4703 (11)	0.0574 (2)	0.0398 (14)	
H20A	0.2444	0.3288	0.0389	0.048*	
C5BA	0.4469 (2)	0.1581 (13)	0.0250 (3)	0.0464 (16)	
H5AA	0.4667	0.0990	0.0111	0.056*	
C23A	0.2477 (2)	0.8811 (13)	0.1134 (3)	0.0479 (16)	
H23A	0.2486	1.0209	0.1324	0.057*	
C17A	0.3387 (2)	0.3144 (11)	0.2077 (2)	0.0351 (13)	
H17A	0.3411	0.3891	0.1788	0.042*	
C21A	0.2097 (2)	0.5140 (13)	0.0718 (2)	0.0433 (14)	
H21A	0.1843	0.4052	0.0619	0.052*	
C19A	0.2827 (2)	0.6336 (11)	0.0698 (2)	0.0338 (13)	
C4BA	0.4804 (2)	−0.1452 (11)	0.0988 (2)	0.0357 (13)	
H4AA	0.5009	−0.1993	0.0854	0.043*	
C14A	0.3307 (2)	0.1026 (12)	0.2930 (3)	0.0421 (15)	
H14A	0.3280	0.0315	0.3221	0.051*	
C22A	0.2115 (2)	0.7196 (11)	0.1009 (2)	0.0370 (14)	
H22A	0.1879	0.7468	0.1119	0.044*	
C6BA	0.4180 (2)	0.3482 (12)	0.0009 (2)	0.0397 (14)	
C2BA	0.4528 (2)	−0.1716 (10)	0.1661 (2)	0.0331 (12)	
H2AA	0.4547	−0.2482	0.1972	0.040*	
C11A	0.3975 (2)	−0.0106 (11)	0.2126 (2)	0.0341 (12)	
H11A	0.4310	−0.0026	0.2422	0.041*	
H11B	0.3885	−0.1783	0.2044	0.041*	
C15A	0.3048 (2)	0.3079 (13)	0.2684 (3)	0.0470 (16)	
H15A	0.2842	0.3741	0.2806	0.056*	
C13A	0.3607 (2)	0.0025 (13)	0.2745 (3)	0.0469 (15)	
H13A	0.3784	−0.1359	0.2911	0.056*	

### Atomic displacement parameters (Å^2^)

**Table:**

	*U*^11^	*U*^22^	*U*^33^	*U*^12^	*U*^13^	*U*^23^
I1BA	0.0530 (3)	0.0509 (3)	0.0396 (2)	0.0078 (2)	0.0138 (2)	0.0003 (2)
I2BA	0.0522 (3)	0.0498 (3)	0.0435 (2)	0.0144 (2)	0.01566 (19)	0.0142 (2)
O2BA	0.030 (2)	0.039 (2)	0.036 (2)	0.0099 (18)	0.0113 (18)	0.0087 (18)
O1BA	0.030 (2)	0.037 (2)	0.037 (2)	0.0072 (18)	0.0116 (17)	0.0099 (18)
C7BA	0.036 (3)	0.034 (4)	0.039 (3)	0.007 (2)	0.014 (3)	0.005 (3)
C1BA	0.028 (3)	0.037 (3)	0.030 (2)	−0.005 (3)	0.011 (2)	−0.001 (3)
C18A	0.030 (3)	0.028 (3)	0.035 (3)	0.005 (2)	0.011 (2)	0.007 (2)
C9BA	0.024 (3)	0.036 (3)	0.032 (3)	−0.001 (2)	0.011 (2)	0.001 (3)
C8BA	0.024 (3)	0.033 (3)	0.027 (3)	−0.003 (2)	0.009 (2)	−0.001 (2)
C3BA	0.030 (3)	0.027 (3)	0.039 (3)	0.000 (2)	0.011 (2)	−0.003 (2)
C12A	0.029 (3)	0.037 (3)	0.029 (3)	0.003 (2)	0.013 (2)	−0.006 (2)
C16A	0.039 (3)	0.045 (4)	0.036 (3)	0.013 (3)	0.011 (3)	−0.003 (3)
C24A	0.021 (3)	0.040 (3)	0.032 (3)	0.001 (2)	0.009 (2)	0.000 (2)
C10A	0.039 (3)	0.042 (4)	0.036 (3)	0.001 (3)	0.018 (3)	0.002 (3)
C20A	0.041 (3)	0.034 (4)	0.041 (3)	0.000 (3)	0.017 (3)	−0.005 (3)
C5BA	0.036 (3)	0.053 (4)	0.044 (3)	0.008 (3)	0.014 (3)	0.003 (3)
C23A	0.046 (4)	0.048 (4)	0.044 (3)	0.014 (3)	0.018 (3)	0.000 (3)
C17A	0.038 (3)	0.034 (3)	0.030 (3)	−0.002 (3)	0.013 (3)	0.000 (2)
C21A	0.033 (3)	0.046 (4)	0.043 (3)	−0.002 (3)	0.012 (3)	−0.002 (3)
C19A	0.033 (3)	0.038 (3)	0.030 (3)	0.007 (3)	0.015 (2)	0.006 (3)
C4BA	0.031 (3)	0.035 (3)	0.041 (3)	0.008 (3)	0.017 (3)	−0.003 (3)
C14A	0.035 (3)	0.047 (4)	0.043 (3)	0.006 (3)	0.018 (3)	0.017 (3)
C22A	0.031 (3)	0.039 (4)	0.036 (3)	0.009 (3)	0.012 (3)	0.007 (3)
C6BA	0.036 (3)	0.047 (4)	0.033 (3)	0.002 (3)	0.015 (3)	0.005 (3)
C2BA	0.030 (3)	0.035 (3)	0.035 (3)	0.001 (2)	0.016 (3)	0.003 (3)
C11A	0.034 (3)	0.037 (3)	0.031 (2)	0.003 (3)	0.015 (2)	0.006 (3)
C15A	0.040 (4)	0.050 (4)	0.043 (3)	0.013 (3)	0.014 (3)	0.002 (3)
C13A	0.046 (3)	0.046 (4)	0.044 (3)	0.024 (3)	0.017 (3)	0.014 (3)

### Geometric parameters (Å, °)

**Table:**

I1BA—C3BA	2.124 (6)	C24A—C23A	1.397 (8)
I2BA—C6BA	2.092 (6)	C24A—H24A	0.9300
O2BA—C8BA	1.361 (6)	C10A—C4BA	1.421 (8)
O2BA—C18A	1.458 (6)	C10A—C5BA	1.425 (8)
O1BA—C1BA	1.369 (7)	C20A—C21A	1.368 (9)
O1BA—C11A	1.434 (6)	C20A—C19A	1.387 (8)
C7BA—C8BA	1.371 (8)	C20A—H20A	0.9300
C7BA—C6BA	1.414 (8)	C5BA—C6BA	1.342 (9)
C7BA—H7AA	0.9300	C5BA—H5AA	0.9300
C1BA—C2BA	1.387 (8)	C23A—C22A	1.354 (9)
C1BA—C9BA	1.439 (7)	C23A—H23A	0.9300
C18A—C19A	1.499 (7)	C17A—H17A	0.9300
C18A—H18A	0.9700	C21A—C22A	1.382 (9)
C18A—H18B	0.9700	C21A—H21A	0.9300
C9BA—C10A	1.430 (8)	C4BA—H4AA	0.9300
C9BA—C8BA	1.433 (8)	C14A—C15A	1.376 (9)
C3BA—C4BA	1.338 (8)	C14A—C13A	1.381 (9)
C3BA—C2BA	1.379 (8)	C14A—H14A	0.9300
C12A—C17A	1.361 (8)	C22A—H22A	0.9300
C12A—C13A	1.410 (8)	C2BA—H2AA	0.9300
C12A—C11A	1.489 (8)	C11A—H11A	0.9700
C16A—C15A	1.345 (9)	C11A—H11B	0.9700
C16A—C17A	1.381 (8)	C15A—H15A	0.9300
C16A—H16A	0.9300	C13A—H13A	0.9300
C24A—C19A	1.380 (8)		
			
C8BA—O2BA—C18A	115.2 (4)	C10A—C5BA—H5AA	119.9
C1BA—O1BA—C11A	116.2 (4)	C22A—C23A—C24A	120.9 (6)
C8BA—C7BA—C6BA	120.7 (5)	C22A—C23A—H23A	119.6
C8BA—C7BA—H7AA	119.6	C24A—C23A—H23A	119.6
C6BA—C7BA—H7AA	119.6	C12A—C17A—C16A	121.5 (6)
O1BA—C1BA—C2BA	122.2 (5)	C12A—C17A—H17A	119.2
O1BA—C1BA—C9BA	116.8 (5)	C16A—C17A—H17A	119.2
C2BA—C1BA—C9BA	121.0 (5)	C20A—C21A—C22A	120.2 (6)
O2BA—C18A—C19A	109.6 (4)	C20A—C21A—H21A	119.9
O2BA—C18A—H18A	109.7	C22A—C21A—H21A	119.9
C19A—C18A—H18A	109.7	C24A—C19A—C20A	118.5 (5)
O2BA—C18A—H18B	109.7	C24A—C19A—C18A	120.4 (5)
C19A—C18A—H18B	109.7	C20A—C19A—C18A	121.0 (5)
H18A—C18A—H18B	108.2	C3BA—C4BA—C10A	119.3 (5)
C10A—C9BA—C8BA	117.7 (5)	C3BA—C4BA—H4AA	120.4
C10A—C9BA—C1BA	116.1 (5)	C10A—C4BA—H4AA	120.4
C8BA—C9BA—C1BA	126.2 (5)	C15A—C14A—C13A	119.8 (6)
O2BA—C8BA—C7BA	123.0 (5)	C15A—C14A—H14A	120.1
O2BA—C8BA—C9BA	116.9 (5)	C13A—C14A—H14A	120.1
C7BA—C8BA—C9BA	120.2 (5)	C23A—C22A—C21A	119.5 (6)
C4BA—C3BA—C2BA	122.9 (5)	C23A—C22A—H22A	120.2
C4BA—C3BA—I1BA	118.7 (4)	C21A—C22A—H22A	120.2
C2BA—C3BA—I1BA	118.4 (4)	C5BA—C6BA—C7BA	121.1 (6)
C17A—C12A—C13A	117.9 (5)	C5BA—C6BA—I2BA	119.3 (5)
C17A—C12A—C11A	125.6 (5)	C7BA—C6BA—I2BA	119.6 (4)
C13A—C12A—C11A	116.5 (5)	C3BA—C2BA—C1BA	119.7 (5)
C15A—C16A—C17A	120.3 (6)	C3BA—C2BA—H2AA	120.1
C15A—C16A—H16A	119.9	C1BA—C2BA—H2AA	120.1
C17A—C16A—H16A	119.9	O1BA—C11A—C12A	108.6 (5)
C19A—C24A—C23A	119.9 (6)	O1BA—C11A—H11A	110.0
C19A—C24A—H24A	120.1	C12A—C11A—H11A	110.0
C23A—C24A—H24A	120.1	O1BA—C11A—H11B	110.0
C4BA—C10A—C5BA	119.0 (6)	C12A—C11A—H11B	110.0
C4BA—C10A—C9BA	121.0 (5)	H11A—C11A—H11B	108.4
C5BA—C10A—C9BA	120.0 (6)	C16A—C15A—C14A	120.5 (6)
C21A—C20A—C19A	121.1 (6)	C16A—C15A—H15A	119.8
C21A—C20A—H20A	119.5	C14A—C15A—H15A	119.8
C19A—C20A—H20A	119.5	C14A—C13A—C12A	120.0 (6)
C6BA—C5BA—C10A	120.2 (6)	C14A—C13A—H13A	120.0
C6BA—C5BA—H5AA	119.9	C12A—C13A—H13A	120.0
			
C11A—O1BA—C1BA—C2BA	−2.1 (8)	C23A—C24A—C19A—C18A	176.4 (5)
C11A—O1BA—C1BA—C9BA	179.4 (5)	C21A—C20A—C19A—C24A	1.1 (9)
C8BA—O2BA—C18A—C19A	174.0 (5)	C21A—C20A—C19A—C18A	−175.3 (5)
O1BA—C1BA—C9BA—C10A	177.3 (5)	O2BA—C18A—C19A—C24A	111.8 (6)
C2BA—C1BA—C9BA—C10A	−1.2 (8)	O2BA—C18A—C19A—C20A	−71.8 (6)
O1BA—C1BA—C9BA—C8BA	−2.7 (8)	C2BA—C3BA—C4BA—C10A	−1.2 (9)
C2BA—C1BA—C9BA—C8BA	178.8 (6)	I1BA—C3BA—C4BA—C10A	−179.0 (4)
C18A—O2BA—C8BA—C7BA	−0.1 (8)	C5BA—C10A—C4BA—C3BA	178.5 (6)
C18A—O2BA—C8BA—C9BA	−179.1 (5)	C9BA—C10A—C4BA—C3BA	1.5 (9)
C6BA—C7BA—C8BA—O2BA	−175.7 (5)	C24A—C23A—C22A—C21A	−1.2 (9)
C6BA—C7BA—C8BA—C9BA	3.3 (9)	C20A—C21A—C22A—C23A	2.2 (9)
C10A—C9BA—C8BA—O2BA	174.3 (5)	C10A—C5BA—C6BA—C7BA	−2.3 (10)
C1BA—C9BA—C8BA—O2BA	−5.7 (8)	C10A—C5BA—C6BA—I2BA	179.0 (5)
C10A—C9BA—C8BA—C7BA	−4.7 (8)	C8BA—C7BA—C6BA—C5BA	0.3 (10)
C1BA—C9BA—C8BA—C7BA	175.3 (5)	C8BA—C7BA—C6BA—I2BA	179.0 (4)
C8BA—C9BA—C10A—C4BA	179.6 (5)	C4BA—C3BA—C2BA—C1BA	−0.4 (9)
C1BA—C9BA—C10A—C4BA	−0.3 (8)	I1BA—C3BA—C2BA—C1BA	177.4 (4)
C8BA—C9BA—C10A—C5BA	2.7 (8)	O1BA—C1BA—C2BA—C3BA	−176.8 (5)
C1BA—C9BA—C10A—C5BA	−177.3 (5)	C9BA—C1BA—C2BA—C3BA	1.7 (8)
C4BA—C10A—C5BA—C6BA	−176.2 (6)	C1BA—O1BA—C11A—C12A	−179.6 (4)
C9BA—C10A—C5BA—C6BA	0.8 (9)	C17A—C12A—C11A—O1BA	−6.4 (8)
C19A—C24A—C23A—C22A	0.2 (9)	C13A—C12A—C11A—O1BA	174.3 (5)
C13A—C12A—C17A—C16A	−0.8 (9)	C17A—C16A—C15A—C14A	1.2 (10)
C11A—C12A—C17A—C16A	180.0 (6)	C13A—C14A—C15A—C16A	−0.8 (10)
C15A—C16A—C17A—C12A	−0.3 (9)	C15A—C14A—C13A—C12A	−0.3 (10)
C19A—C20A—C21A—C22A	−2.2 (9)	C17A—C12A—C13A—C14A	1.1 (9)
C23A—C24A—C19A—C20A	−0.1 (8)	C11A—C12A—C13A—C14A	−179.6 (6)

### Hydrogen-bond geometry (Å, °)

**Table:**

Cg is the centroid of the C19A–C24A ring.

**Table:**

*D*—H···*A*	*D*—H	H···*A*	*D*···*A*	*D*—H···*A*
C24A—H24A···O1BA^i^	0.93	2.49	3.348 (8)	154
C18A—H18A···Cg^ii^	0.97	2.77	3.513 (5)	134

Symmetry codes: (i) *x*, *y*+1, *z*; (ii) −*x*+1/2, −*y*+3/2, −*z*.
